# DGrA: Lightweight Modulation Recognition Based on Hybrid Neural Networks

**DOI:** 10.3390/s26103259

**Published:** 2026-05-21

**Authors:** Xu Chen, Rui Gao, Ding Xu, Hongbo Zhu

**Affiliations:** 1 Jiangsu Key Laboratory of Wireless Communications, Nanjing University of Posts and Telecommunications, Nanjing 210003, China; 2018010203@njupt.edu.cn (X.C.); xuding@njupt.edu.cn (D.X.); zhuhb@njupt.edu.cn (H.Z.); 2 School of Information Engineering, Yangzhou University, Yangzhou 225000, China

**Keywords:** modulation recognition, deep learning, lightweight, SDR, intelligent signal processing, feature extraction

## Abstract

Automatic modulation recognition has been recognized as an effective technique for non-cooperative communication and intelligent transmission. In this paper, we propose a new lightweight method for automatic modulation recognition, aiming to extract crucial discriminative features of signals for higher recognition accuracy while reducing spatial costs. To enhance the dissimilarity between samples, this paper combines an improved attention block and convolutional operations with the recurrent neural network, focusing on key features during the training phase to efficiently differentiate signal sequences. By replacing standard convolutions with depthwise separable convolutions, the model’s computational complexity is reduced while enhancing its feature extraction capability. Furthermore, the method incorporates pruning to reduce ineffective features, decreasing the model size while maintaining performance. Experimental results on RadioML2016.10a demonstrate that the proposed method outperforms other comparative methods, exhibiting both higher recognition accuracy and smaller model size. To validate real-world applicability, the algorithm was implemented on a software-defined radio platform for signal transmission and reception under practical conditions, achieving an accuracy of 87.22% in the presence of environmental noise, thus confirming its effectiveness in real-world scenarios.

## 1. Introduction

Automatic modulation recognition (AMR) aims to automatically identify the modulation scheme from received unknown signals [1]. Driven by the increasing complexity of modern communication systems, AMR serves as a critical enabler for modern applications [2,3]. For instance, in cognitive radio, AMR is employed by secondary users to blindly identify the modulation of primary users, enabling dynamic spectrum access without explicit signaling. In military and civilian spectrum monitoring, this technique will be widely employed to identify malicious interference and ensure physical-layer security. Thus, AMR finds wide applications in various military and civilian sectors [4], including implementation in Internet of Things (IoT) [5], fifth-generation (5G), and beyond-5G wireless systems [6,7,8]. Traditional modulation recognition methods can be primarily categorized into likelihood-based (LB) methods [9] and feature-based (FB) methods [10,11]. LB modulation recognition methods require prior knowledge to analyze and derive the statistical characteristics of received signals theoretically, obtaining test statistics that are compared with appropriate thresholds for decision-making [12]. FB methods, on the other hand, extract feature parameters from received signals and determine the modulation type through pattern recognition systems.

With the widespread application of deep learning (DL) [13,14] in artificial intelligence fields such as image recognition and natural language processing, numerous DL-based AMR algorithms have been proposed [15], which directly input preprocessed received signals into neural networks for training and recognition. In [16], contrastive learning is used for modulation recognition. The whole process requires little prior knowledge, greatly surpassing traditional methods. Early studies [17,18] tended to convert radio signals into images, constructing recognition models based on convolutional neural networks (CNNs) that distinguish modulation mode features by extracting local patterns [19]. In [20], the clustering algorithm is used to extract and optimize the characteristic parameters of the signal to improve the recognition performance under low signal-to-noise ratio (SNR). By continuously extracting temporal features, methods based on recurrent neural networks (RNNs) [21] can outperform those based on CNNs. Furthermore, by integrating CNNs and RNNs [22] to fully utilize temporal and spatial information, recognition performance can be improved [23,24,25]. Although these methods can achieve state-of-the-art accuracy, they still require a large number of parameters, which makes it difficult to balance performance and complexity.

The deployment of lightweight AMR models on resource-constrained edge devices is of great practical importance. Nevertheless, among these DL-based AMR methods, there have been few attempts to combine attention blocks and depthwise separable convolution (DSC) [26] simultaneously. Large recognition models make embedded deployment difficult; to solve this problem, a lightweight AMR method is proposed, namely depthwise separable convolution-assisted gated recurrent units with improved attention blocks (DGrA). This method effectively reduces model size while ensuring recognition performance. Specifically, in complex wireless channels, signals are inevitably corrupted by noise and multi-path fading, making critical features difficult to capture. To address this, the RNN combined with an improved attention block is designed. The attention mechanism dynamically assigns higher weights to the most discriminative temporal and spatial features while suppressing irrelevant noise, thereby providing a strong rationale for enhancing the model’s feature focusing capability. In order to reduce the training parameters of the network and further extract sequential feature information, DSCs are employed as a substitute for regular convolutions. Additionally, pruning is applied to reduce the model size. The main contributions of this work are summarized as follows.

- We propose a feasible method by utilizing the focusing capability of improved attention blocks. Additionally, by replacing standard convolutions with DSCs, the proposed model preserves the spatial characteristics of signals while reducing computational complexity. The GRU module is further employed to capture sequential dependencies in the signal.
- To further reduce the network size while ensuring recognition performance, pruning is employed in combination with the AMR method, making it more suitable for lightweight deployment. A series of experiments are conducted to evaluate the performance of the proposed model. Experimental results demonstrate that combining the improved attention blocks and DSCs can effectively enhance the recognition accuracy of the model, and the proposed model has significant advantages in lightweight deployment.
- To validate the algorithm’s effectiveness under real-world channel conditions, we developed a ZYNQ-based modulation recognition system using a software-defined radio (SDR) platform. The implemented system enables wireless signal transmission and reception in practical channel environments. Through comprehensive evaluation with various modulation schemes, the proposed lightweight algorithm demonstrates robust recognition performance that meets practical requirements in authentic channel scenarios.

The rest of the paper is organized as follows. Section 2 describes the signal model. The proposed DGrA model is presented in Section 3. Section 4 gives simulation experiments, and Section 5 concludes the paper.

## 2. Signal Model

Typically, the received signal [21] in wireless communication systems can be represented as

(1) $$y\left(t\right)=h\left(t\right)*M\left(s(t)\right)+w\left(t\right),$$

where M(·) is the modulation function, s(t) is the transmitted symbol, ∗ denotes the convolution operator, h(t) is the channel effect, w(t) denotes the additive white Gaussian noise (AWGN), and y(t) is the unknown modulated signal at the receiver. The received signal can be preprocessed into I/Q sequences as

(2) y(n)=ℜ{y(n)}+jℑ{y(n)},n=1,2,…,N,

where ℜ{y(n)} and ℑ{y(n)} represent the real and imaginary parts of the signal, respectively, $j=\sqrt{-1}$, and *N* is the sample length. To facilitate data processing and modulation recognition, the received signal can be further represented as

(3) $$Y=\left[\begin{array}{c} y_{i}\\ y_{q} \end{array}\right]=\left[\begin{array}{c} ℜ\{y(1)\},ℜ\{y(2)\},\ldots ,ℜ\{y(N)\}\\ ℑ\{y(1)\},ℑ\{y(2)\},\ldots ,ℑ\{y(N)\} \end{array}\right],$$

where $y_{i}$ and $y_{q}$ represent the in-phase and quadrature components, respectively.

**Figure 1:**
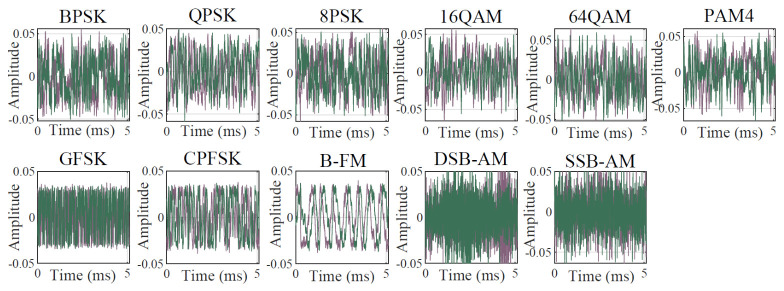
Waveforms of different signals. The dark purple and green curves denote the in-phase and quadrature components, respectively.

**Figure 2:**
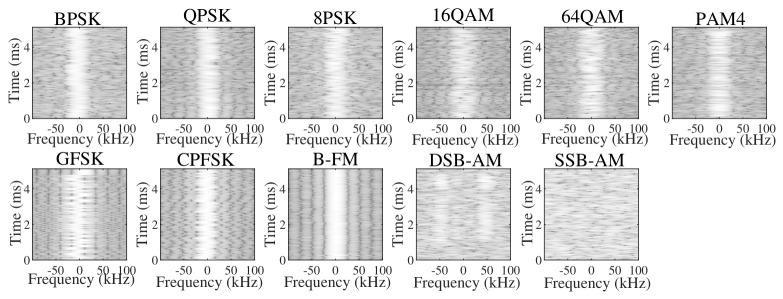
Spectrograms of different signals.

In this paper, 11 types of signals sampled from the open-source RadioML2016.10a dataset are used. These signals are generated by passing ideal modulated symbols through simulated practical wireless channels, incorporating additive white Gaussian noise, multi-path fading, and frequency offsets. Figure 1 and Figure 2 depict the resulting waveforms and spectrograms. It is evident that recognizing these signals is highly challenging. First, high-order modulation schemes exhibit extreme similarity in both time and frequency domains. Second, the severe waveform distortion caused by low signal-to-noise ratio environments makes accurate feature extraction extremely difficult for conventional algorithms.

## 3. Proposed DGrA Model

**Figure 3:**
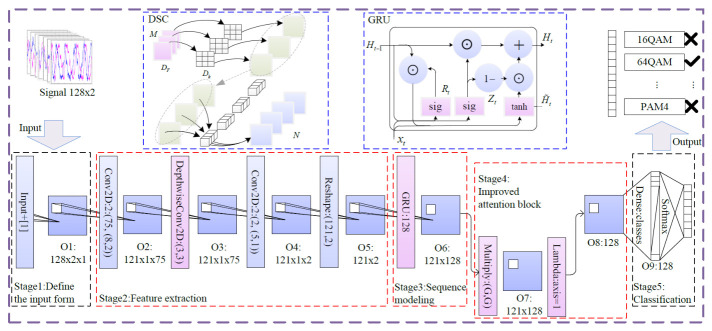
The structure of the proposed DGrA AMR model.

Recognition accuracy and model size are two important criteria in AMR tasks. However, conventional methods often overlook the latter. To address this problem and achieve better performance, a novel AMR method based on an RNN is proposed by combining improved attention blocks and DSCs. The enhanced network optimizes the model size. The proposed framework is depicted in Figure 3.

Incorporating convolutions in AMR tasks proves beneficial. However, traditional CNN- and RNN-based AMR methods reintroduce a significant number of parameters, leading to an increase in model size. To achieve lightweight models, regular convolutions are replaced with DSCs. Simultaneously, embedding improved attention blocks enables the extraction of crucial features, thereby enhancing the overall performance of the network. Moreover, to meet the requirements of lightweight AMR models, pruning is employed to further reduce the model size.

### 3.1. Modified Network Model

The steps of the proposed DGrA model are given as follows:

**Stage 1: Define the input format.** The generated signal data consists of I/Q signals with 128 points for each channel. Therefore, the shape of the input feature map is (128, 2). In CNNs, input data typically have three dimensions: width, height, and the number of channels. To facilitate convolutional processing, the input data format is expanded to (128, 2, 1), representing each sample with 128 time steps. Each time step contains 2 features, and the number of channels is 1.
**Stage 2: Feature extraction.** The convolutional layer uses 75 filters, with a kernel size of (8, 2) and rectified linear unit (ReLU) activation function. The input feature map is multiplied and summed element-wise with the convolutional kernel to obtain a scalar value. The convolutional kernel then moves along the height and width dimensions of the input, repeating this operation to generate the output feature map. The dimensions of the output feature map from the convolutional layer are derived based on the sliding-window mechanism. Given VALID padding, which indicates that no zero-padding is applied at the edges, the convolution kernel can only be placed at positions where it fully overlaps with the input feature map. For an input height $H_{i}$, kernel height $H_{k}$, and stride *S*, the output height is calculated as

(4) $$H_{o}=\left\lfloor \frac{H_{i}-H_{k}}{S}\right\rfloor +1,$$

and the output width is calculated as

(5) $$W_{o}=\left\lfloor \frac{W_{i}-W_{k}}{S}\right\rfloor +1,$$

where $W_{i}$ and $W_{k}$ denote the input width and kernel width, respectively, and ⌊·⌋ represents the floor function.

The kernel size of DSC is (3, 3) and the stride is 1. The DSC decomposes the convolution operation into two separate steps: depth-wise convolution and point-wise convolution. The depth-wise convolution reduces the number of parameters by decreasing the number of convolutional filters. The point-wise convolution is designed to transform the low-dimensional feature maps generated by the depth-wise convolution into higher-dimensional feature representations. The DSC does not change the height and width, and the output height and width remain consistent with the input height. In the DSC, each input channel is convolved element-wise with the corresponding depth-wise convolutional filter to generate the corresponding feature map. As the input channel depth is 75, the output channel depth is also 75, indicating that each input channel corresponds to the generation of a feature map, and the output depth is the same as the input depth. DSC reduces the number of parameters and computational operations, thereby making the model more lightweight and efficient.

Further utilizing convolutional layers, there are two filters with a kernel size of (5, 1) and stride = 1. When padding = SAME, the size of the output feature map is determined by

(6) $$H_{o}=\mathrm{ceil}\left(H_{i}/S\right),$$

(7) $$W_{o}=\mathrm{ceil}\left(W_{i}/S\right),$$

where $H_{i}=121$, $H_{o}=121$. $W_{i}=1$, and $W_{o}=1$. The depth of the output feature map is equal to the number of convolutional filters, which is 2 in this case. The convolutional layer is added to project the output of the DSC into a new representation. The output tensor is then reshaped to (121,2) for subsequent sequence modeling. The mathematical operation can be expressed as $X_{out}=\mathrm{ReLU}\left(W*X_{in}+b\right)$. Functionally, the depthwise separable convolution effectively extracts spatial local correlations within the I/Q streams while significantly reducing the parameter count compared to standard convolutions.

**Stage 3: Sequence modeling.** The hidden state size of the GRU network is 128, returning the outputs of all time steps for sequence modeling. The hidden state update is mathematically described as $h_{t}=\mathrm{GRU}\left(x_{t},h_{t-1}\right)$. Functionally, the GRU is specifically employed to capture the long-term temporal dependencies and phase variations inherent in the modulated signal sequences.
**Stage 4: Improved attention block.** Let the output sequence of the final GRU layer be denoted as $H\in R^{T\times D}$, where *T* represents the time steps and *D* represents the hidden state size. The sequence is element-wise multiplied with the computed attention weights α. Specifically, the output of the final GRU layer is multiplied element-wise to obtain the weighted feature representation $H_{w}=H⊙\alpha$. This mathematical technique is utilized to adaptively highlight crucial features in the sequence and suppress the impact of noise.

Subsequently, the weighted results are aggregated by summing along the sequence dimension. Mathematically, the final attention-weighted output vector $v\in R^{D}$ is calculated as $v=\sum_{t=1}^{T}H_{w}\left(t,:\right)$. We specify axis=1 in the implementation to indicate this summation process along the time steps, producing a compact and discriminative feature vector for the subsequent classification.

**Stage 5: Classification.** A dense layer is added for classification with an output size of classes=11, representing the number of classes. The activation function used is softmax.

### 3.2. Pruning Operation

Pruning achieves model compression by reducing unimportant parameters in the model. This paper adopts a weight-based pruning method, which determines the importance of parameters based on their magnitude and performs pruning operations accordingly. Weight sparsification can be used during training, and the specific formula is as follows.

(1) **Pruning rule:** First, set a global pruning ratio. Based on this ratio, a portion of the minimum absolute value elements in the weight matrix are set to zero.
(2) **Pruning operation:** For each weight matrix W, calculate its $L_{1}$-norm ${∥W∥}_{1}$,
  (8) $${∥W∥}_{1}=\sum \left|w_{ij}\right|,$$
  where $w_{ij}$ represents the element at the *i*-th row and *j*-th column of the weight matrix W.
(3) **Normalizing weights and setting threshold:** Divide each element in the weight matrix W by its corresponding $L_{1}$-norm to obtain the normalized weight matrix $W^{\prime }$,
  (9) $$W^{\prime }=W/{∥W∥}_{1}.$$
  Calculate the pruning threshold T based on the global pruning ratio according to the pruning rule
  (10) $$T=\alpha *\mathrm{maximum}\left({∥W∥}_{1}\right),$$
  where α is a parameter used to control the pruning ratio, and $\mathrm{maximum}\left({∥W∥}_{1}\right)$ represents the maximum $L_{1}$-norm of the weight matrix W.
(4) **Pruning operation:** For the normalized weight matrix $W^{\prime }$, set the elements with absolute values smaller than the threshold T to zero
  (11) $$W^{\prime \prime }=W^{\prime }*\left(\left|W^{\prime }\right|\geq T\right),$$
  where $\left|W^{\prime }\right|\geq T$ represents setting the elements with absolute values greater than or equal to the threshold T to 1. Otherwise, they are set to 0.

Applying this pruning method during the training process involves gradually pruning the weights of the model through iterative optimization. The pruned model can exhibit sparsity, reducing storage and computational requirements while potentially improving the model’s generalization capability. To clearly illustrate the computational steps, the standard pruning operation is summarized in Algorithm 1.
**Algorithm 1** Weight-based Pruning Process**Require:** Initial weight matrix *W*, global pruning ratio α
**Ensure:** Pruned weight matrix $W^{\prime \prime }$
  1:**Initialize:** Train the model to obtain the unpruned weight matrix *W*.  2:**Calculate Norm:** Compute the $L_{1}$-norm of the weight matrix: ${∥W∥}_{1}=\sum \left|w_{ij}\right|$  3:**Normalize:** Obtain the normalized weight matrix: $W^{\prime }=W/{∥W∥}_{1}$  4:**Set Threshold:** Calculate pruning threshold: $T=\alpha \times \max(∥W{∥}_{1})$  5:**Apply Mask:**  6:**for** each element $w_{ij}^{\prime }$ in $W^{\prime }$ **do**  7:    **if** $|w_{ij}^{\prime }|\geq T$ **then**  8:        $w_{ij}^{\prime \prime }\leftarrow w_{ij}^{\prime }$  9:    **else**10:        $w_{ij}^{\prime \prime }\leftarrow 0$11:    **end if**12:**end for**13:**Return:** Sparse model with updated weights $W^{\prime \prime }$


## 4. Experiments and Results

In this section, experiments are conducted to evaluate the recognition performance of the proposed model. To begin with, the dataset and training parameters used are introduced. Subsequently, the recognition performance of different neural network models under different SNRs is compared, validating the superior recognition performance of the proposed model. Finally, a performance comparison is conducted before and after pruning.

### 4.1. Experimental Setup and Dataset

The open-source dataset RadioML2016.10a [27] is adopted for training in this paper. The dataset used in the simulations contains 11 modulation types: 8PSK, BPSK, CPFSK, GFSK, 4PAM, 16QAM, 64QAM, QPSK, AM-DSB, AM-SSB, WBFM. The specific parameters are shown in Table 1. To simulate real wireless environments, factors such as noise, fading, sampling rate offset, and center frequency offset are taken into account during dataset generation. The total size of the dataset is 220,000 × 2 × 128. While this dataset primarily contains classical modulation schemes, it is utilized in this study because it serves as the most widely adopted standard benchmark in the AMR literature, ensuring a fair and consistent comparison of model parameters and accuracy against existing state-of-the-art methods.

All neural-network models were implemented in Python (v3.8.10) using TensorFlow (v2.6.0) and Keras (v2.6.0). The training and evaluation scripts were executed on a workstation equipped with an NVIDIA GPU using CUDA (v11.2) and cuDNN (v8.1). The training parameters are shown in Table 2. The specific parameters of the proposed network are listed in Table 3. A pruning ratio of 0.2 is set, which means 80% of the parameters in the model are retained.

### 4.2. Simulation Results

Table 4 compares the performance of the proposed method from different perspectives. To facilitate comparison, several DL-based AMR models are implemented, including LSTM [21], GRU [22], ResNet [28], GRU-Att, Cov-GRU-Att, Cov-GRU-SAt, and DGrA. The highest recognition accuracy achieved by the proposed method is 91.1%, with a compressed model size of 611464 bytes. It can be observed that DGrA not only achieves the highest recognition accuracy but also has the smallest model size. This is because the proposed method builds upon the GRU model by adding DSCs and improved attention blocks. In this study, the criterion of recognition accuracy is defined mathematically as the ratio of correctly predicted signal frames to the total number of tested signal frames in the dataset, formulated as $\mathrm{Accuracy}=\left(\frac{N_{\mathrm{correct}}}{N_{\mathrm{total}}}\right)\times 100\%$.

It is important to note that although the absolute accuracy improvement over the Cov-GRU-SAt model is modest, the primary advantage of the proposed DGrA lies in its lightweight efficiency. Specifically, DGrA reduces the compressed model size from 785,177 bytes to 611,464 bytes, corresponding to a reduction of more than 22%, while maintaining competitive recognition accuracy. This result indicates that the proposed architecture can reduce redundant parameters while preserving recognition performance.

**Figure 4:**
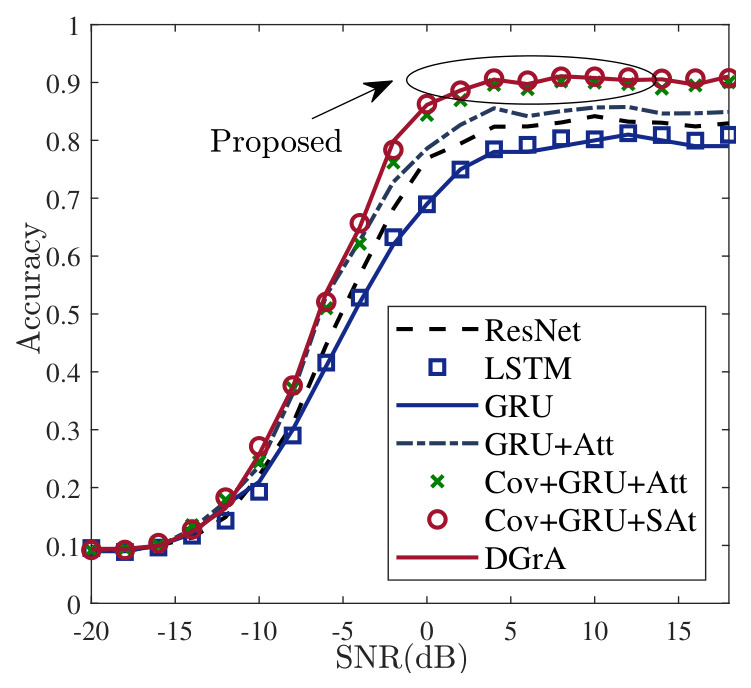
Comparison of recognition accuracy at all SNRs.

Figure 4 shows the accuracy curves of different methods at various SNRs. It is evident that the proposed DGrA model consistently outperforms other models. This is because the model is able to capture both temporal and spatial features from the dataset and pays more attention to the discriminative features of the signals. Although Cov-GRU-SAt and the proposed method have similar recognition performance, according to Table 4, the proposed method has fewer parameters, which is closer to the requirements of lightweight models. Regarding computational complexity, Table 4 shows that the proposed DGrA achieves an optimized spatial footprint, with only 54,807 parameters and a compressed model size of 611,464 bytes, making it more lightweight than other high-accuracy models such as Cov-GRU-SAt. Although depthwise separable convolution reduces the number of parameters and the theoretical computational cost, the practical training time is also affected by memory access overhead, framework-level kernel optimization, and the sequential nature of GRU and attention operations. Therefore, DGrA is particularly suitable for storage-constrained edge devices. In terms of noise robustness, DGrA maintains an average accuracy of 60.9% across the full SNR range from −20 dB to 18 dB, indicating strong resilience to severe noise. This robustness benefits from the improved attention mechanism, which emphasizes key structural signal features while suppressing noise-induced anomalies.

The implications of these results suggest that the proposed DGrA is highly suitable for embedded deployment in edge-computing IoT devices where hardware resources are strictly constrained. However, a notable shortcoming is the persistent confusion between 16QAM and 64QAM. This indicates that the current temporal window length of 128 samples might be insufficient to fully capture the statistical differences of dense constellation points, and real-world high-speed mobility scenarios were not fully addressed.

**Figure 5:**
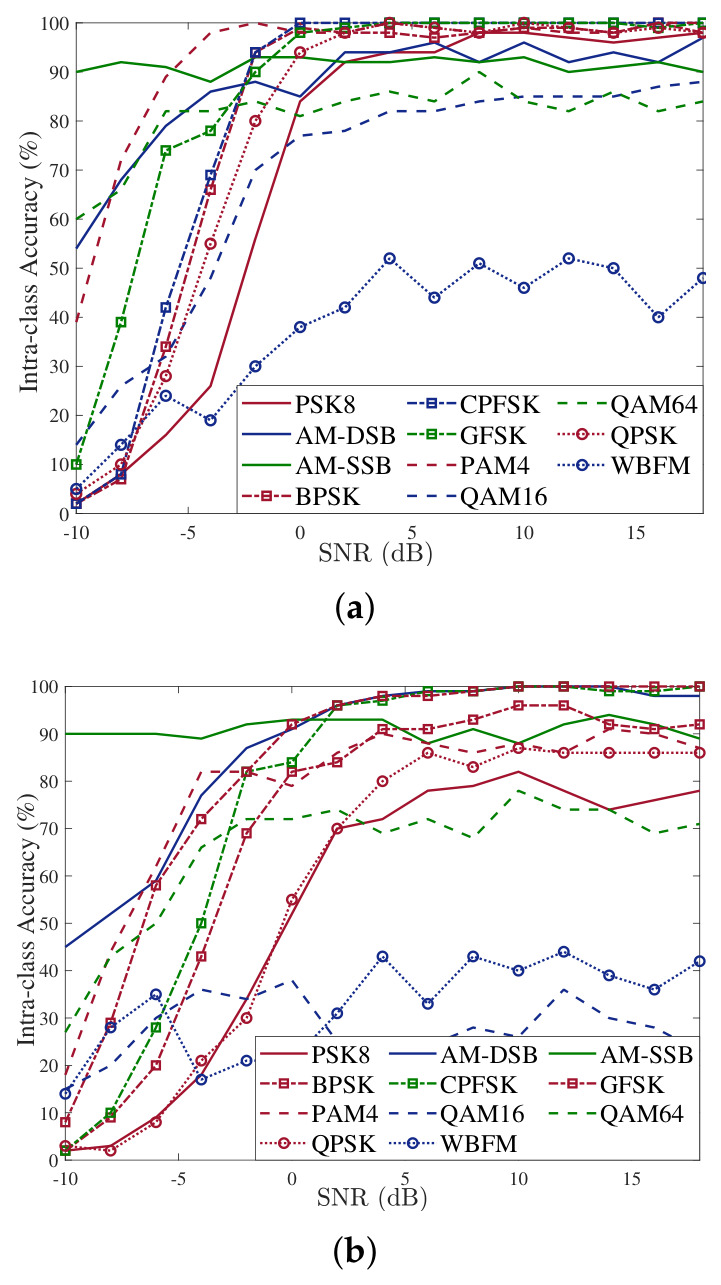
Intra-class recognition accuracy of the proposed model and GRU. (**a**) Intra-class recognition accuracy of the proposed model. (**b**) Intra-class recognition accuracy of GRU.

Figure 5 presents the intra-class recognition rate curves of the proposed method and the GRU-based modulation recognition model. It can be observed that after the improvements, there is a significant increase in the recognition rate for each modulation scheme at different SNRs. Although the simulation plots include SNR values below −5 dB, where recognition accuracy is inevitably low due to severe noise corruption, this low-SNR region is retained to evaluate the performance boundary of the models under extreme noise conditions. In practical applications, our primary focus is on the operational region above −2 dB, where the proposed DGrA model begins to exhibit clear performance advantages over the baseline methods.

**Figure 6:**
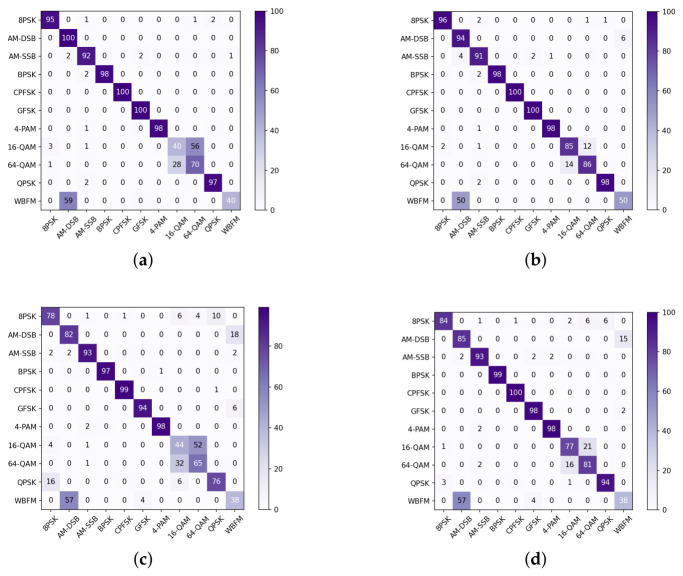
Confusion matrices of the proposed model and GRU-Att. (**a**) Confusion matrix of GRU-Att with SNR = 14 dB. (**b**) Confusion matrix of DGrA with SNR = 14 dB. (**c**) Confusion matrix of GRU-Att with SNR = 0 dB. (**d**) Confusion matrix of DGrA with SNR = 0 dB.

Figure 6 compares the confusion matrix of GRU-Att and DGrA at the same SNR. When the SNR is 14 dB, the proposed model has an overall relatively high recognition accuracy, almost fully extracting the effective components of the signal and effectively distinguishing the modulation signals. It can be observed that 16QAM is easily misclassified as 64QAM. This is because the waveform of 16QAM is similar to that of 64QAM in the time domain. When the SNR drops to 0 dB, the recognition performance is lower, but the proposed model still has a relatively high recognition accuracy for CPFSK, GFSK, BPSK, and 4PAM.

**Figure 7:**
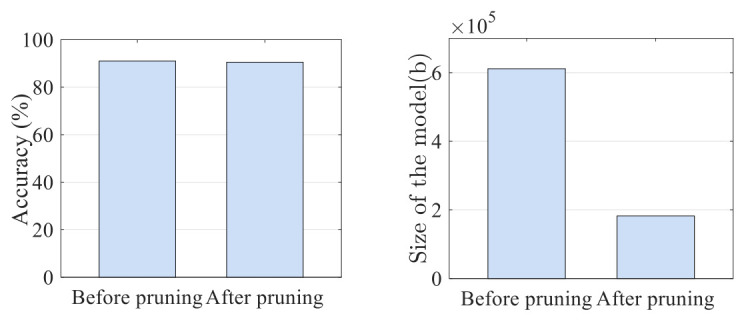
The performance comparison of the proposed model before and after pruning.

In Figure 7, a bar graph is plotted to illustrate the performance comparison before and after pruning of the proposed method. Adopting the pruning method sacrificed approximately 1% of the recognition rate while reducing the model size by approximately one-third. Evidently, there is minimal change in the recognition rate before and after pruning, but the size of the model is significantly reduced. Hence, the pruned model is considered as the optimal model. Table 5 summarizes the key results and practical implications of the proposed DGrA model.

### 4.3. Hardware Implementation

**Figure 8:**
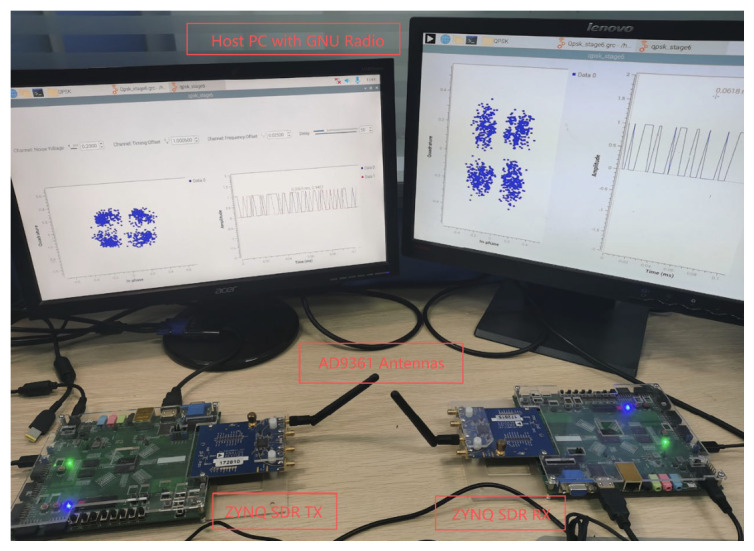
Experimental scenario deployment.

This study establishes a wireless signal transceiver system utilizing two software-defined radios (SDRs) to validate the effectiveness of the proposed lightweight DGrA network for modulation recognition in practical channels. As illustrated in Figure 8, the experimental configuration employs ZYNQ-based SDR platforms equipped with AD9361 RF transceiver chipsets as transmitter and receiver units. The AD9361 RF transceiver was sourced from Analog Devices Inc. (Wilmington, MA, USA), and the ZYNQ system-on-chip was sourced from Xilinx Inc. (San Jose, CA, USA). These SDR systems were implemented through GNU Radio (v3.10.7.0). The experiment was conducted in a typical wireless communication channel environment without physical obstructions between transceivers. During the measurements, we assumed a quasi-static channel where the environment remained stable during the transmission of each frame. It is important to note the limitations of the measurement equipment: the AD9361 chipset has a limited baseband bandwidth and may introduce minor local oscillator leakage and I/Q imbalance, which are typical in low-cost SDRs and present realistic hardware impairments for the recognition model.

Notably, signal propagation through this channel exhibits characteristic phenomena including reflection, scattering, and path loss. Both the transmitter and receiver were configured in stationary positions with no high-speed motion involved, thereby rendering Doppler frequency shift effects negligible in this experimental scenario. Furthermore, the GNU Radio platform configured in the SDR systems facilitates real-time signal transmission and reception. To obtain an approximately 8 dB SNR in the real SDR system, the ambient noise floor was first measured by the receiving SDR without any transmission. Subsequently, the transmission gain of the TX SDR was precisely calibrated until the received signal power was 8 dB above the measured noise floor. The 8 dB SNR was specifically selected because, as observed in our simulation results, it represents a critical transitional point where models approach their maximum accuracy, making it a suitable testing condition to validate the alignment between real-world performance and theoretical simulations.

We generated training waveforms through the following procedure. For each modulation scheme, 10,000 frames were created with a 60%–20%–20% split for training, validation, and testing, respectively. During network training, both training and validation frames were utilized, while the test set was exclusively employed for final classification accuracy evaluation. Each frame contains 1024 samples acquired at a 200 kHz sampling rate, maintaining consistent configuration parameters across all training and validation phases.

**Figure 9:**
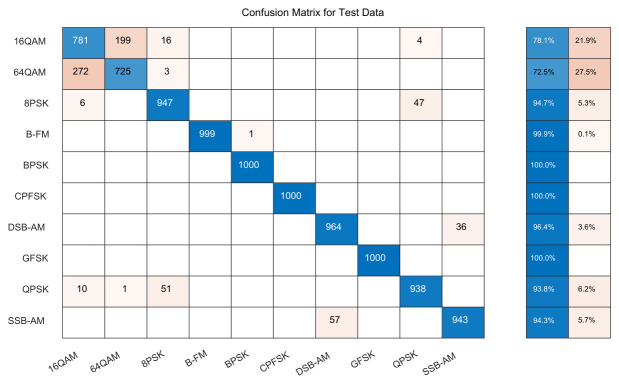
Confusion matrix of the test signals. The color bar represents the normalized classification proportion.

To comprehensively evaluate the performance of the established network model, we constructed a confusion matrix using signals from the test set, as depicted in Figure 9. Matrix analysis reveals an overall recognition accuracy of 92.54%, demonstrating the proposed lightweight DGrA network’s effectiveness and efficiency in modulation recognition tasks. The results indicate particular challenges in distinguishing between 16QAM- and 64QAM-modulated signals. This observed confusion exhibits a systematic nature rather than random occurrence, which can be theoretically attributed to the fact that 16QAM constitutes a subset of 64QAM when considering each signal frame contains only 128 symbols. This inherent relationship consequently introduces differentiation difficulties for the network between these two modulation schemes.

**Figure 10:**
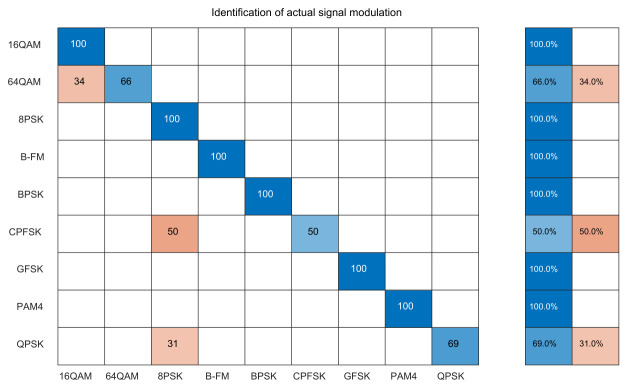
Confusion matrix of the actual signals. The color bar represents the normalized classification proportion.

To further verify the performance of the modulation recognition algorithm proposed in this paper under actual communication conditions, the trained model was saved, and a setup using ZYNQ-based SDR platforms equipped with AD9361 RF transceivers as transmitter and receiver, as shown in Figure 8, was constructed. The transmitter and receiver were placed 60 cm apart. The transmitter sent the original signals through a channel containing natural environmental noise, which were then captured in real time by the receiver and subjected to modulation signal identification. This study employed a lightweight neural network model to conduct recognition experiments on modulation signals collected by the software-defined radio. The confusion matrix, presented in Figure 10, shows that the model achieved a 100% recognition accuracy for 16QAM, WBFM, GFSK, PAM4, and QPSK. The overall recognition rate under natural environmental noise was 87.22%, indicating extremely high recognition performance for these modulation schemes. For 64QAM and CPFSK, the recognition accuracies were 66% and 50%, respectively, indicating certain errors in practical signal modulation recognition. These errors may stem from the signal-to-noise ratio, the similarity of modulation schemes, or the model’s inadequate learning of specific modulation type characteristics. Despite these misclassifications, the overall performance substantiates the practical viability of the lightweight neural network approach for real-world modulation recognition tasks.

## 5. Conclusions

In this paper, a novel and efficient lightweight AMR method is proposed. The key strategy is the redesign of the network architecture, incorporating improved attention modules that enhance the discriminative ability of the network towards crucial signal features. Subsequently, DSC is employed to reduce the model size. Furthermore, pruning is applied to achieve lightweight models, ensuring both accuracy and reduced size in the proposed method. The experimental results demonstrate that the proposed method outperforms others in terms of recognition accuracy and model size, making it more suitable for lightweight deployment in communication applications. The experimental validation employs a heterogeneous computing architecture enabling cross-platform interoperability and real-time processing. Through multi-domain signal characterization combining time–frequency analysis and constellation diagram features, we developed an SDR-based recognition system validated under practical channel conditions. The framework achieves 87.22% recognition accuracy in noisy environments, with perfect classification for 16QAM, WBFM, GFSK, PAM4, and QPSK modulations. Real-time implementation on embedded SDR platforms demonstrates inference performance, confirming practical deployment capability in resource-constrained systems. Future work will focus on expanding the proposed framework to recognize modern complex modulation schemes, such as OFDM and Chirp signals, to further meet the demands of next-generation communication systems.

## Figures and Tables

**Table 1:** Simulation and dataset parameters.

Parameters	Value
SNR range	−20 dB:2 dB:18 dB
Data points per frame	1024
Points per symbol	8
Sampling rate	200 kHz
Total number of signal frames	220,000
Noise	Additive White Gaussian Noise

**Table 2:** Specific training parameters.

Hyperparameters	Value
Optimizer	Adam
Maximum training epochs	300
Batch size	400
Loss function	Cross entropy
Training set: Validation set: Test set	6:2:2
Sparsity of pruning	0.2

**Table 3:** Parameters of the network.

Layer	Input Size	Output Size
Input: input shape	(128, 2)	(128, 2, 1)
Conv2D: (75, (8, 2))	(128, 2, 1)	(121, 1, 75)
DepthwiseConv2D: (3, 3)	(121, 1, 75)	(121, 1, 75)
Conv2D: (2, (5, 1))	(121, 1, 75)	(121, 1, 2)
Reshape: target shape = (121, 2)	(121, 1, 2)	(121, 2)
GRU: 128	(121, 2)	(121, 128)
Multiply: (GRU output, GRU output)	(121, 128)	(121, 128)
Lambda: axis = 1	(121, 128)	128
Dense: classes	128	11

**Table 4:** Performance comparison of different methods.

Name of Model	ResNet	LSTM	GRU	GRU-Att	Cov-GRU-Att	Cov-GRU-SAt	DGrA
Highest recognition accuracy (%)	82.9	80.9	78.6	84.9	90.1	90.7	**91.1**
Total parameter size	3,098,283	68,491	52,107	52,236	71,743	71,614	**54,807**
H5 model size (KB)	36,371	833	641	656	899	886	**694**
Compressed model size (bytes)	31,517,698	774,195	589,834	590,236	782,269	785,177	**611,464**
−20 dB–18 dB average accuracy	54.8	52.2	51.9	57.4	59.9	61.1	**60.9**
Average time (s/epoch)	43.6	6.8	5.5	6.9	7.1	6.6	**7.7**
Actual training epoch	137	259	245	171	106	96	**104**
Total training time (s)	5967.1	1764.3	1344.1	1173.4	750.1	637.4	**804.9**

Note: Bold values indicate the results of the proposed DGrA model.

**Table 5:** Summary of key results and implications of the proposed DGrA model.

Evaluation Metric	Achieved Value
Highest recognition accuracy	91.1%
Average accuracy over all SNRs	60.9%
Compressed model size	611,464 bytes
Number of parameters	54,807
Real-world SDR accuracy	87.22%
Main limitation	16QAM/64QAM confusion

## Data Availability

Publicly available datasets were analyzed in this study. The open-source dataset RadioML2016.10a can be found here: https://www.deepsig.ai/datasets (accessed on 7 May 2026).
